# Methylophiopogonanone A Inhibits Ferroptosis in H9c2 Cells: An Experimental and Molecular Simulation Study

**DOI:** 10.3390/molecules29235764

**Published:** 2024-12-06

**Authors:** Yanqing Wang, Xi Zhao, Ban Chen, Shaoman Chen, Yongbai Liang, Dongfeng Chen, Xican Li

**Affiliations:** 1 Department of Anatomy, School of Basic Medical Sciences, Guangzhou University of Chinese Medicine, Guangzhou 510006, China; yanqingwang@stu.gzucm.edu.cn; 2 School of Chinese Herbal Medicine, Guangzhou University of Chinese Medicine, Guangzhou 510006, China; jjy199406180220@163.com (X.Z.); 20221110152@stu.gzucm.edu.cn (S.C.); 20231110153@stu.gzucm.edu.cn (Y.L.); 3 School of Life and Health Sciences, Hubei University of Technology, Wuhan 430068, China; chenban@hbut.edu.cn

**Keywords:** homoisoflavone, ferroptotic inhibition, H9c2 cell line, molecular docking simulation, molecular dynamics simulation

## Abstract

In this study, homoisoflavone methylophiopogonanone A (MOA) was investigated for its inhibitory effect on ferroptosis of H9c2 cells using a set of cellular assays, such as BODIPY-probed and H_2_DCFDA-probed flow cytometry analyses, cell counting kit-8 analysis (CCK-8), and lactate dehydrogenase (LDH) release analysis. All these cellular assays adopted Fer-1 as the positive control. Subsequently, MOA and Fer-1 were subjected to two antioxidant assays, i.e., 2-phenyl-4,4,5,5-tetramethylimidazoline-1-oxyl 3-oxide radical (PTIO^•^)-scavenging and 2,2′-azinobis(3-ethylbenzo-thiazoline-6-sulfonic acid radical (ABTS^•+^)-scavenging. Finally, MOA, along with Fer-1, were systematically analyzed for molecular docking and dynamics simulations using a set of software tools. The experimental results revealed that MOA could inhibit ferroptosis of H9c2 cells but did not effectively scavenge PTIO^•^ and ABTS^•+^ free radicals. Two molecular simulation methods or algorithms suggested that MOA possessed similar binding affinity and binding free energy (∆G_bind_) to Fer-1. Visual analyses indicated various hydrophobic interactions between MOA and one of the seven enzymes, including superoxide dismutase (SOD), dihydroorotate dehydrogenase (DHODH), ferroportin1 (FPN), ferroptosis suppressor protein 1 (FSP1), glutathione peroxidase 4 (GPX4), nicotinamide adenine dinucleotide phosphate (NADPH), and solute carrier family 7 member 11 (SLC7A11). Based on these experimental and molecular simulation results, it is concluded that MOA, a homoisoflavonoid with *meta*-di-OHs, can inhibit ferroptosis in H9c2 cells. Its inhibitory effect is mainly attributed to the regulation of enzymes rather than direct free radical scavenging. The regulation of enzymes primarily depends on hydrophobic interactions rather than H-bond formation. During the process, flexibility around position 9 allows MOA to adjust to the enzyme binding site. All these findings provide foundational information for developing MOA and its derivatives as potential drugs for myocardial diseases.

## 1. Introduction

Ferroptosis, an iron-dependent form of regulated cell death [1], has become increasingly recognized as an important process that mediates the pathogenesis and progression of numerous cardiovascular diseases, including atherosclerosis [2], heart failure [3], myocardial ischemia–reperfusion injury [4], sepsis-induced cardiomyopathy [5], arrhythmia [6], and diabetic cardiomyopathy [7]. Correspondingly, various synthetic and natural small molecules have been screened as ferroptosis inhibitors [8,9].

**Figure 1:**
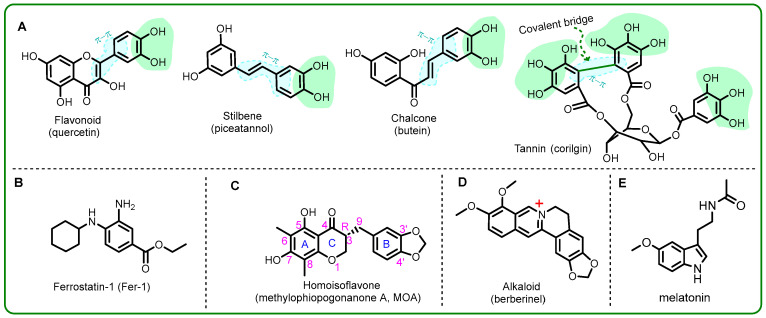
Structures of small molecules with ferroptosis inhibition or antioxidant potential. (**A**) Natural antioxidants from the previous studies; (**B**) natural antioxidant in the present study; (**C**) synthetic ferroptosis inhibitors in both the present and previous studies; (**D**) natural non-antioxidant from the previous study; (**E**) natural antioxidant and ferroptosis inhibitor from the previous study.

Some ferroptosis inhibitors are natural antioxidants, such as flavonoid [8], stilbene [10], chalcone [11], corilagin [12], and tannin [13,14] (Figure 1A). From the perspective of chemical biology, one common feature among them is the presence of catechol or pyrogallol moieties. These moieties have been reported to possess strong antioxidant abilities via free radical scavenging [8,14]. Another feature is the occurrence of exocyclic π–π conjugation, which may facilitate the delocalization of π-electrons and, thus, stabilize the intermediate radical after the scavenging reaction (Figure 1A). As a result, exocyclic π–π is considered to enhance the effectiveness of natural antioxidants [15,16]. Particularly, tannin has advantages in free radical scavenging and showed extraordinary inhibitory effects on ferroptosis. Corilagin, a covalent bridging tannin, is a typical example. The covalent bridge in the corilagin molecule could twist the chair conformation to elevate the molecular energy to effectively scavenge free radicals (especially reactive oxygen species, ROS) [12]. All these findings suggest that a natural ferroptosis inhibitor is equivalent to a natural antioxidant.

However, the suggestion is challenged by ferrostatin-1 (Fer-1, Figure 1B), an effective ferroptosis inhibitor that lacks free radical scavenging abilities [13]. This means that other factors must facilitate the inhibition of ferroptosis. To investigate these factors, a homoisoflavone, methylophiopogonanone A (MOA), was evaluated in this study for the presence of three specific structures. As shown in Figure 1C, its B-ring and C-ring are blocked by a –CH_2_– (at the 9-position); two phenolic –OH groups are located at the meta-position (i.e., the 5-position and the 7-position) rather than ortho-position. Additionally, the B-ring contains a 3′,4′-methylenedioxyl group, which can form phenolic –OH esterfication.

The introduction of MOA is also supported by pharmacological evidence. Previous investigations have revealed that MOA has a variety of pharmacological effects, including anti-inflammatory properties and the ability to inhibit apoptosis in myocardial cells and improve of cerebral ischemia/reperfusion injuries [17,18,19]. Considering the substantial clinical significance of ferroptosis and its inhibition in the H9c2 cell line within the myocardial field [20,21,22,23], H9c2 was selected as the cell line model to explore the potential inhibitory effect of MOA on ferroptosis.

In the H9c2 cell model, erastin was used as the ferroptotic inducer. Subsequently, the effectiveness of MOA as a ferroptosis inhibitor was measured using flow cytometry with 4′,6-diamidino-2-phenylindole (DAPI) staining, a H_2_DCFDA probe, a C11-BODIPY probe, and a CCK-8 solution. Moreover, the role of MOA’s specific structure in interacting with ferroptosis-related enzymes was investigated through molecular docking simulations. These enzymes include acyl-CoA synthetase long-chain family member 4 (ACSL4), dihydroorotate dehydrogenase (DHODH), divalent metal transporter 1 (DMT1), ferroportin1 (FPN), ferroptosis suppressor protein 1 (FSP1), glutathione peroxidase 4 (GPX4), ferritin heavy chain 1 (FTH1), nicotinamide adenine dinucleotide phosphate (NADPH), nuclear factor erythroid 2-related factor 2 (NRF2), solute carrier family 7 member 11 (SLC7A11), superoxide dismutase (SOD), and transferrin receptor protein 1 (TFR1) [24,25,26,27,28,29,30]. This study aims to enhance our understanding of alternative approaches to inhibit ferroptosis beyond typical antioxidation. It will also contribute to the development of MOA as a drug candidate for the treatment of myocardial diseases.

## 2. Results and Discussion

The apoptosis or survival of H9c2 cells plays an important role in the field of cardiology. Laynes’s team reported that cardiotoxicity arises from the activation of apoptosis in the H9c2 cell line. This apoptosis results from mitochondrial H_2_O_2_ and related ROS accumulation, ultimately leading to cytotoxicity in cardiomyocytes [31,32]. Ferroptosis, a form of regulated cell death, can also occur in H9c2 cells, and has been shown to contribute to myocardial injury, according to a recent study [23]. The present study aimed to quantitatively determine the relative ROS inhibition level of MOA in H9c2 cells. Therefore, flow cytometry analysis based on C11-BODIPY staining was employed, using Fer-1 (1 µM) as the positive control due to its effectiveness in inhibiting ferroptosis [33].

**Figure 2:**
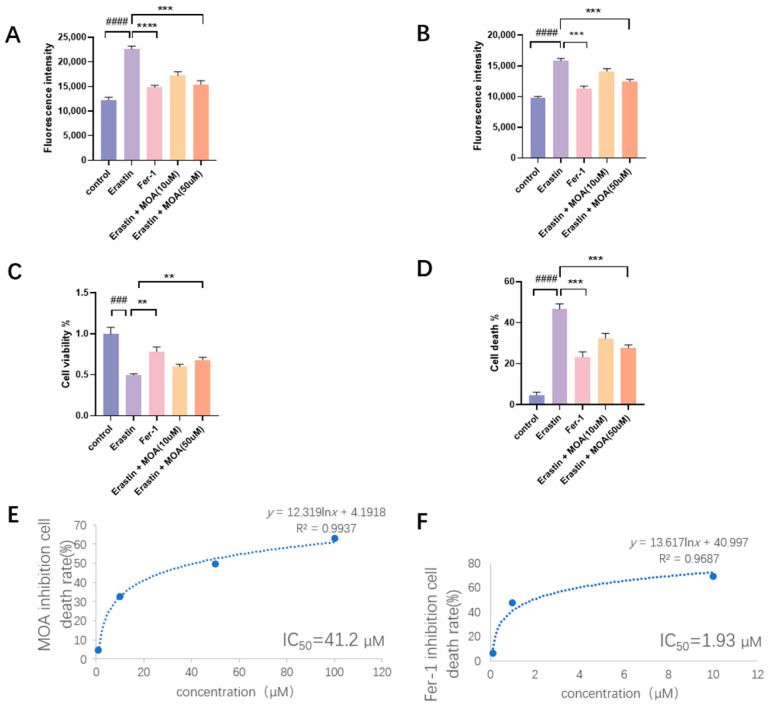
The main results of cellular experiments for the inhibitory effect on ferroptosis of MOA. (**A**) BODIPY-probed flow cytometry analysis; (**B**) H_2_DCFDA-probed flow cytometry analysis; (**C**) cell counting kit-8 (CCK-8) analysis; (**D**) lactate dehydrogenase (LDH) release analysis. (**E**,**F**) The dose–response curves and IC_50_ values of MOA and Fer-1 in the LAD release analysis. H9c2 cells treated with 10 µM of erastin in the presence of 1–100 µM MOA (**E**); H9c2 cells treated with 10 µM of erastin in the presence of 0.1–10 µM Fer-1 (**F**). Control group: H9c2 cells without erastin treatment; erastin group: 10 µM of erastin-treated H9c2 cells; Fer-1 group: H9c2 cells treated with 10 µM of erastin + 1 µM Fer-1; MOA 10 µM group: H9c2 cells treated with 10 µM of erastin + 10 µM MOA; MOA 50 µM group: H9c2 cells treated with 10 µM of erastin + 50 µM MOA. Data are presented as means ± SDs from three independent experiments. ### *p* < 0.001, #### *p* < 0.0001, compared with the control group; ** *p* < 0.01, *** *p* < 0.001, **** *p* < 0.0001 compared with the erastin group.

As shown in Figure 2, the positive control Fer-1 displayed the highest level of inhibition. At a concentration of 50 µM, MOA was observed to exhibit significant inhibition (Figure 2). Lipid peroxidation (LPO) is known to be transformed from other ROS (such as ^•^O_2_^−^ [34] and ^•^OH [35]). Thus, the total ROS levels in the cells were further measured using H_2_DCFDA-based flow cytometry. The results showed that MOA reduce ROS levels in a dose-dependent manner (Figure 2B). The inhibition of LPO and other ROS has been reported to improve cell viability [36]. Accordingly, MOA enhanced cell viability in a dose-dependent manner (Figure 2C). Correspondingly, MOA reduced cell death percentages in a dose-dependent manner (Figure 2D). Accordingly, the IC_50_ values of MOA and Fer-1 were calculated to be 41.2 µM (Figure 2E) and 1.93 µM (Figure 2F), respectively.

**Figure 3:**
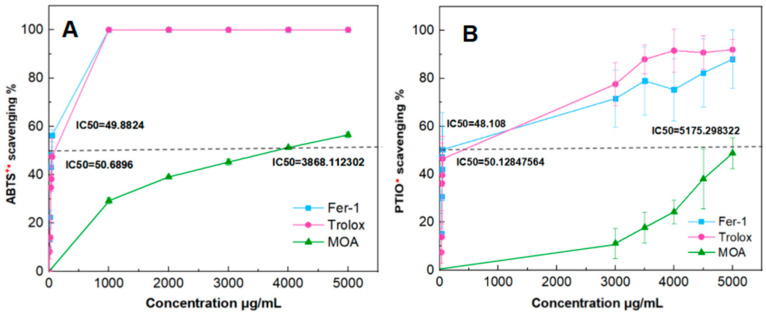
The dose–response curves and IC_50_ values of MOA, Fer-1, and Trolox in two antioxidant assays. (**A**) ABTS^•+^-scavenging assay; (**B**) PTIO^•^-scavenging assay. (Each value was expressed as a mean ± SD, n = 3).

To investigate whether the inhibitory effect on ferroptosis of MOA results from direct free radical scavenging, PTIO^•^-scavenging and ABTS^•+^-scavenging assays were conducted in this study. As shown in Figure 3A,B, MOA exhibited 77 times and 103 times higher IC_50_ values than a positive control of Trolox in the PTIO^•^-scavenging and ABTS^•+^-scavenging assays, respectively. This means that MOA, as a ferroptosis inhibitor, showed a very low direct free radical scavenging level in both an aqueous medium and a lipid medium. This can be attributed to the absence of an *ortho*-di-OHs group (i.e., catechol moiety), which is present in tannin corilagin (Figure 1A).

As mentioned above, the two –OH groups of MOA are *meta*- to each other (see Figure 1C). Our previous study indicated that *meta*-OHs had a very weak antioxidant ability. This is because the oxidized product of *meta*-di-OHs is either a *semi*-quinone radical or a *meta*-benzoquinone. Both of these species are highly unstable [37,38].

These have basically denied the possibility that MOA undergoes direct free radical scavenging to exert its ferroptosis inhibition action. Therefore, its ferroptosis inhibition can be mainly attributed to its interaction with ferroptosis-related enzymes. In fact, MOA could regulate SOD to inhibit the production of ROS and the release of LDH [39]. Such enzyme regulate effect can explain the results of BODIPY-probed flow cytometry and H_2_DCFDA-probed flow cytometry analyses, where MOA was observed to show substantial ROS inhibition levels (Figure 2A,B).

Besides SOD, another eight enzymes were also reported to be closely associated with ferroptosis or ferroptosis inhibition, i.e., DHODH, FPN, FSP1, GPX4, FTH1, NADPH, Nrf2, and SLC7A11 [27,40,41,42,43,44,45,46,47,48,49].

Therefore, an experiment was conducted with all nine enzymes in a molecular docking simulation (Table 1). It was found that NRF2 and FTH1 could not be effectively bound by MOA. Meanwhile, seven ferroptosis inhibition enzymes could be effectively bound by MOA, and their binding affinities by MOA varied from −10.1~−4.0 kcal/mol, while those by Fer-1 ranged from −8.1~−4.7 kcal/mol.

**Figure 4:**
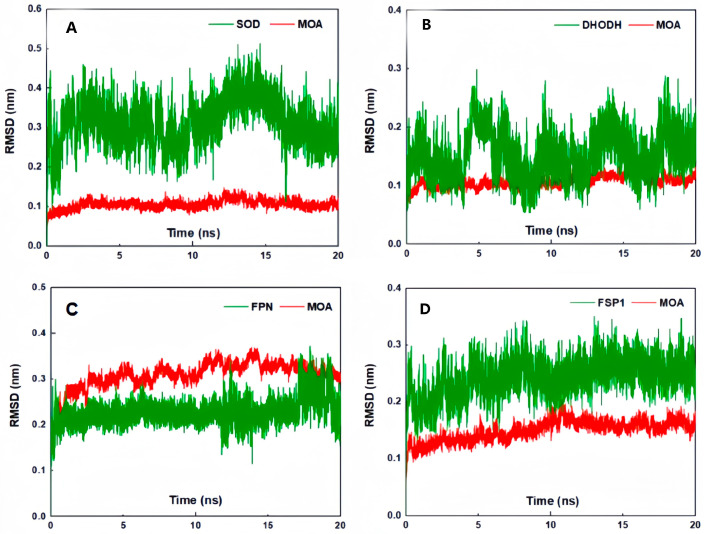

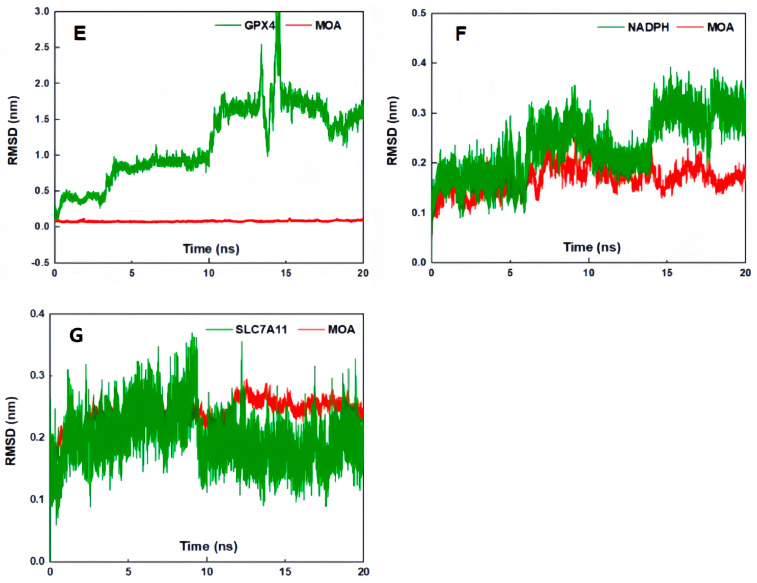
Results of the RMSD analysis obtained from the molecular dynamics simulation. The binding of (**A**) MOA to SOD; (**B**) MOA to DHODH; (**C**) MOA to FPN; (**D**) MOA to FSP1; (**E**) MOA to GPX4; (**F**) MOA to NADPH; and (**G**) MOA to SLC7A11. The simulation was conducted using the Gromacs 2020.6 package, and the results were plotted using Origin 2017 software.

**Figure 5:**
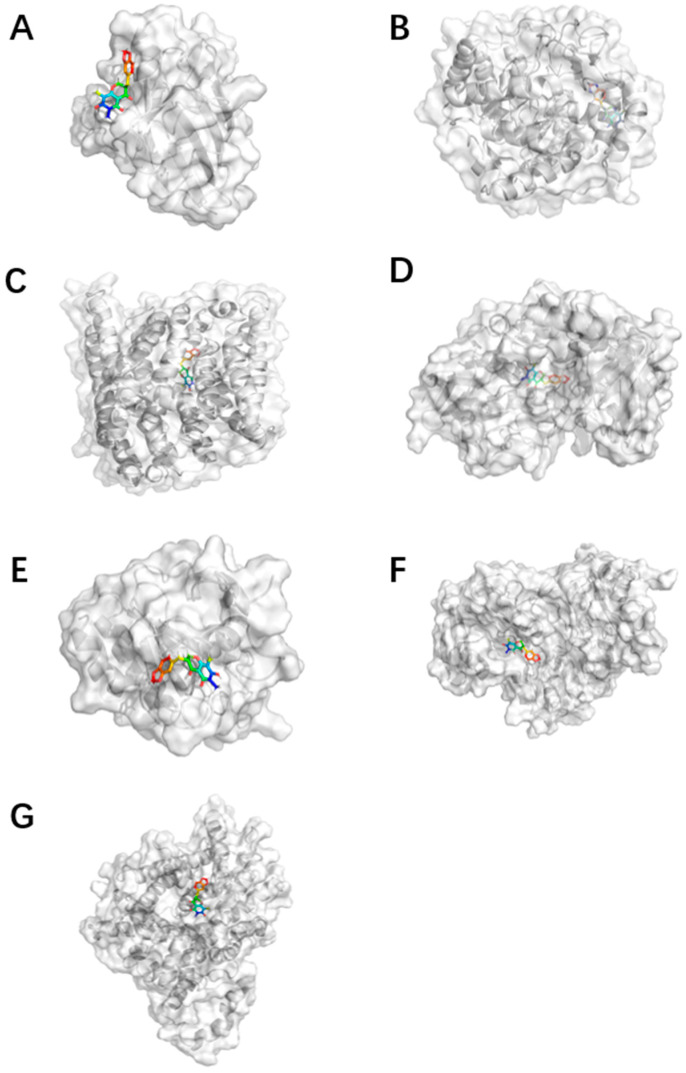
Construction of pharmacophore models using the MOA binding and complex structure. The binding of (**A**) MOA to SOD; (**B**) MOA to DHODH; (**C**) MOA to FPN; (**D**), MOA to FSP1; (**E**) MOA to GPX4; (**F**) MOA to NADPH; (**G**) MOA to SLC7A11. The simulation was conducted using the Gromacs 2020.6 package.

Furthermore, a molecular dynamics simulation was also performed in the study. The root-mean-square error (RMSD) results were visualized to provide a detailed explanation of the dynamic processes of MOA and Fer-1 interactions with different enzymes (Figure 4). The RMSD of each ligand was found to be maintained within a fluctuation of 0.1 nm over a simulation time of 20 ns, which implies that the ligands did not undergo significant conformational changes upon interaction with the enzymes. However, the degree of fluctuation of the enzymes was much more significant than that of the ligands, with most of them showing oscillating or increasing RMSD values from 0 to 15 ns until the last 5 ns, when they stabilized in the range of 0.1 nm. The stabilizing trend in the last 5 nm of RMSD indicates that the ligand and receptor have bound to each other; thus, the structures after their combination are shown in Figure 5. Based on the trajectory in the last 5 ns, the binding free energy ($\Delta G_{\mathrm{bind}}$) was further calculated using molecular mechanics with the generalized Born and surface area solvation (MM/PBSA) method.

As seen in Table 2, the $\Delta G_{\mathrm{bind}}$ values of Fer-1 were in a range between −94.80 ± 2.61 and 1.34 ± 3.90 kcal/mol, while those of MOA varied from −30.63 ± 2.03 to −3.82 ± 1.23 kcal/mol. In particular, the MOA ligand showed a lower $\Delta G_{\mathrm{bind}}$ value than the positive control, Fer-1, when interacting with SOD and GPX4. In a word, the results of binding free energy ($\Delta G_{\mathrm{bind}}$) were slightly different from those of binding affinity in the molecular docking simulation. Nevertheless, it is obvious that both MOA and Fer-1 could effectively bind seven ferroptosis inhibition enzymes. The binding energy ($\Delta G_{\mathrm{bind}}$) is composed of various terms, with the bonding terms (i.e., $\Delta E_{bond}$, $\Delta E_{angle}$, and $\Delta E_{dihedral}$) all being zero, indicating the absence of chemical bonding between the ligand and the receptor. The non-bonding terms, $\Delta E_{ele}$ and $\Delta E_{vdW}$, are typical contributors of H-bond and hydrophobic interactions, respectively, and they contributed significantly to $\Delta G_{\mathrm{bind}}$ in the present study. These findings suggest that H-bond and hydrophobic interactions play a role in MOA or Fer-1 enzyme interactions.

**Figure 6:**
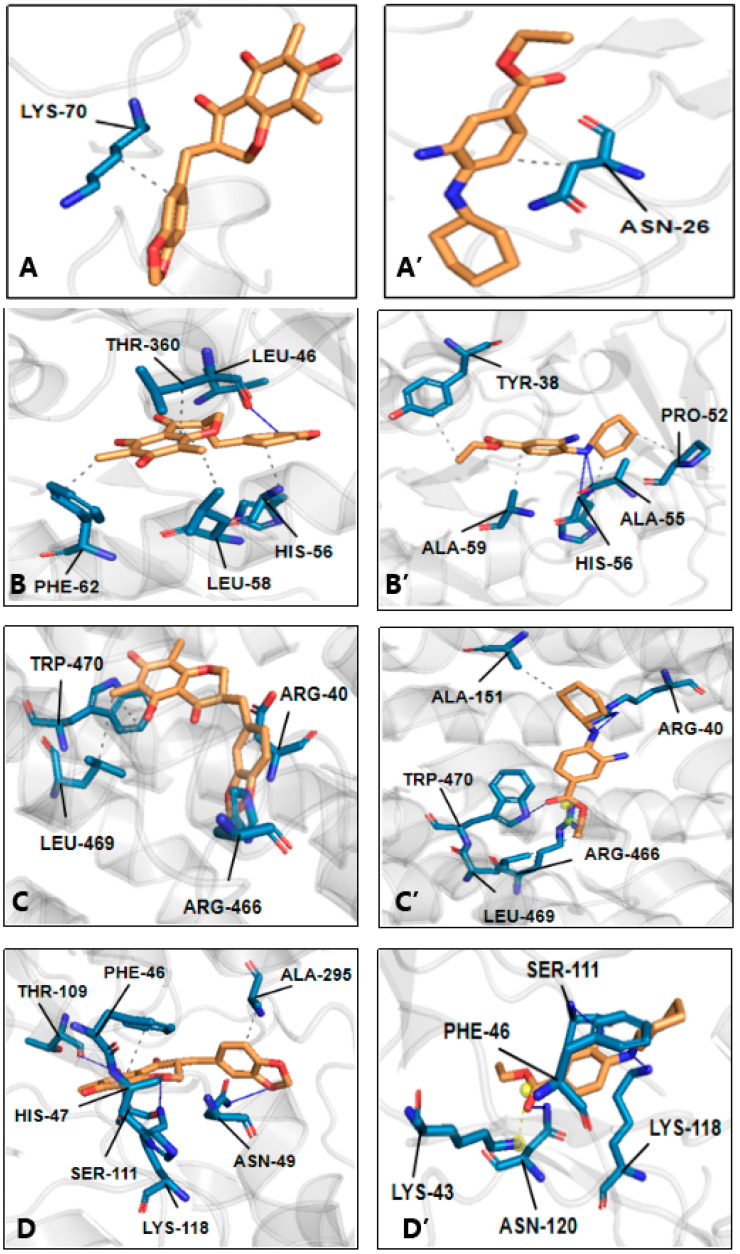

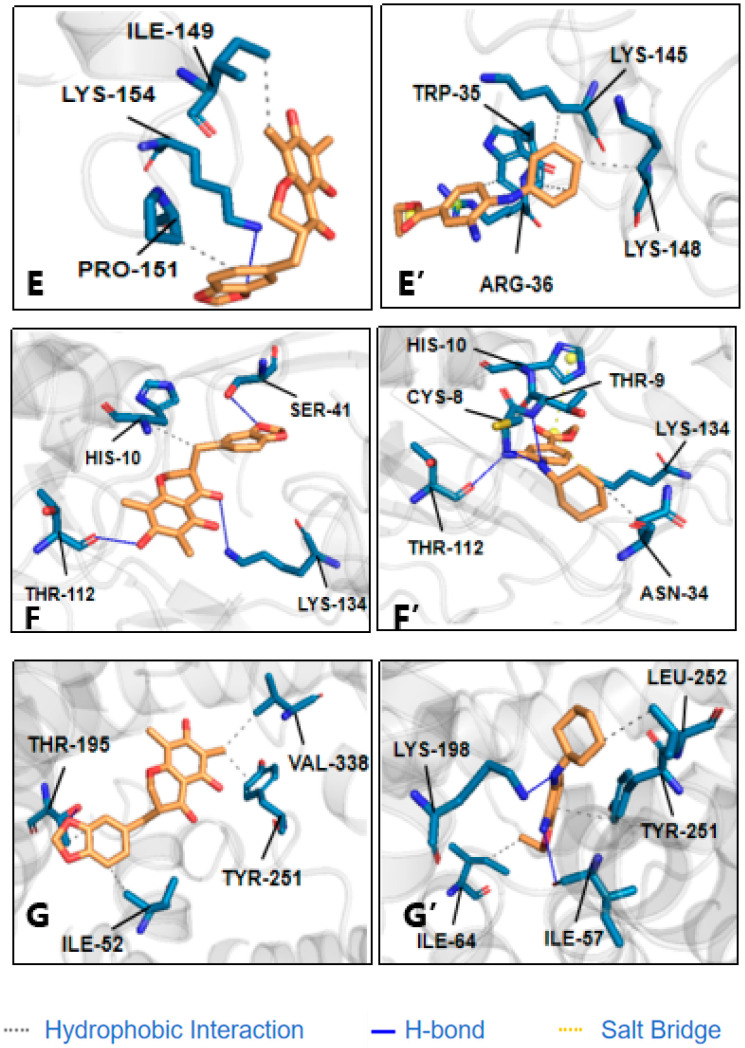
Visualized results of the interactions of methylophiopogonanone (MOA) and Fer-1 with the amino acid residues of seven ferroptosis enzymes. The binding of (**A**) MOA to SOD; (**B**) MOA to DHODH; (**C**) MOA to FPN; (**D**) MOA to FSP1; (**E**) MOA to GPX4; (**F**) MOA to NADPH; (**G**) MOA to SLC7A11. (**A’**), Fer-1 to SOD; (**B’**), Fer-1 to DHODH; (**C’**), Fer-1 to FPN; (**D’**), Fer-1 to FSP; (**E’**), Fer-1 to GPX4; (**F’**), Fer-1 to NADPH; (**G’**), Fer-1 to SLC7A11. The molecular dynamics simulation was conducted using Gromacs 2020.6 package.

As shown in Figure 6, in the binding of MOA, there were 8 H-bonds and 17 hydrophobic interactions, while there were 8 H-bonds and 18 hydrophobic interactions in the binding of Fer-1. The formation of H-bonds requires the presence of active H. Herein, the so-called “active H” is actually a hydrogen atom bearing a partial positive charge (δ^+^) that can be considered a proton.

If the active H (especially a proton) is from the MOA ligand, it may donate it to an enzyme to undergo various pathways to finish its antioxidant action. This is, of course, accompanied by electron transfer. According to the sequence of electron transfer, these antioxidant pathways could be classified into four types, i.e., proton-coupled electron transfer (PCET), sequential electron proton transfer (SEPT), proton loss single electron transfer (SPLET), and hydrogen atom transfer (HAT) [50,51]. Therefore, the formation of H-bonds is closely associated with antioxidation. Of course, the premise is that the active H must be from a ligand, e.g., MOA.

As seen in Figure 1, this kind of active H actually occurred in the 5-OH and 7-OH of the MOA molecule; however, only 7-OH formed one H-bond with THR 112 (Figure 6F). This suggests that antioxidation plays a minor role in the binding enzyme of MOA. The suggestion is further supported by the fact that both Trolox and Fer-1 showed strong radical scavenging abilities (Figure 2); however, Trolox possessed much weaker ferroptosis-inhibitory abilities than Fer-1 [52]. In a word, MOA would be unlikely to directly scavenge free radicals (especially ROS) to inhibit ferroptosis in H9c2 cells. This can be attributed to the 5,7-di-OHs group of MOA that sits at the *meta*-site and cannot produce a stable oxidized product, as mentioned above.

Therefore, MOA had to rely on hydrophobic interactions to bind enzymes. Many cells (e.g., H9c2 cells) are stabilized in an aqueous medium because massive H_2_O molecules will squeeze hydrophobic groups (e.g., –CH_3_, –OCH_3_, –CH_2_–, and even –C_6_H_5_) in amino acids. These hydrophobic groups thereby combine with each other to construct a small hydrophobic region. When an MOA ligand enters this region, it will also be squeezed by H_2_O molecules, accessing the above hydrophobic groups and constructing a so-called “hydrophobic interaction”. The driving force of H_2_O molecule squeezing is the incompatibility between polar H_2_O molecules and non-polar hydrophobic groups.

In fact, in the MOA molecule, there are several non-polar hydrophobic groups, including the A-ring, C-ring, 6–CH_3_, 8–CH_3_, 9–CH_2_–, and 3′,4′-*methylenedioxyl* group. As seen in Figure 6 (left), most of these hydrophobic interactions are connected to these hydrophobic groups. Binding to SOD is a typical example, where the sole hydrophobic interaction was targeted onto the C-ring of MOA (Figure 6A). Another typical example is the binding of SOD to DHODH, which utilizes its amino acids to interact with the 6–CH_3_, A-ring, and C-ring (Figure 6B).

In addition, the non-polar group 9–CH_2_– also forms a hydrophobic interaction with HIS 10 in NADPH (Figure 6F). The chemical bonds in the –CH_2_– group are well-known to be σ-bonds, which are constructed using *sp*^3^-C atoms and show a specific tetrahedron configuration. As a result, all the chemical bonds in the group are rotatable. Thus, the –CH_2_– group could rotate the σ-bond to adjust the atom (or atomic group) in order to access the amino acid residues of enzymes. For example, in the binding of MOA to FPN (Figure 6C), the access of two amino acids (ARG 40 and ARG 466) to the C-ring relied on the rotation of a σ-bond in the –CH_2_– group to a great extent. Similar instances were also observed in Figure 6C–E,G.

## 3. Materials and Methods

### 3.1. Chemicals and Biological Kits

MOA (Cas. 74805-92-8, C_19_H_18_O_6_, M.W. 342.34, Lot No: HO160892) was obtained from Herbest Biotech Co., Ltd. (Baoji, China). Erastin (Cas. 571203-78-6) was obtained from MedChemExpress (Monmouth Junction, NJ, USA). Ferrostatin-1 (Fer-1, Cas. 347174-05-4, M.W. 262.35) was purchased from Selleck Chemicals (Houston, TX, USA). (±)-6-Hydroxyl-2,5,7,8-tetramethylchromane-2-carboxylic acid (Trolox) and (NH_4_)_2_ABTS (2,2′-azinobis(3-ethylbenzo-thiazoline-6-sulfonic acid diammonium salt)) were obtained from Amresco Chemical Co. (Solon, OH, USA). Water and methanol were of HPLC grade. Other reagents of analytical grade were purchased from Guangdong Guanghua Chemical Plants Co., LTD. (Shantou, China). H9c2 cells, fetal bovine serum (FBS), and trypsin were obtained from Molecular Probes (Carlsbad, CA, USA). The C11-BODIPY probe was purchased from Molecular Probes (Carlsbad, CA, USA). The annexin V/propidium iodide (PI) determination kit was purchased from BD Biosciences (Franklin Lakes, NJ, USA). The cell counting kit-8 was purchased from the Dojindo Chemistry Research Institute (Kumamoto, Japan).

### 3.2. Flow Cytometry for the Assessment of LPO Accumulation Using a C11-BODIPY Probe

H9c2 cells were assessed for their LPO accumulation [24]. In brief, the H9c2 cells were seeded at 1 × 10^6^ cells/well into 12-well plates. After 24 h of culturing, the H9c2 cells were classified into a control group, erastin group, erastin plus Fer-1 group, and sample group, as mentioned in the literature [53]. In the control group, the H9c2 cells were incubated for 12 h in basal medium. In the erastin and sample groups, the H9c2 cells were incubated with the mixture of 10 µM of erastin and a sample at different concentrations. In the erastin plus Fer-1 group, the H9c2 cells were incubated with a mixture of 10 µM of erastin and 1 µM of Fer-1. After incubation for 12 h, the mixture of erastin and medium was discarded. The erastin and erastin plus Fer-1 groups were incubated for 12 h in the above basal medium, while the sample groups were incubated for 12 h in the above basal medium with the sample. Following incubation, the cells were rinsed with PBS two times. The H9c2 cells interacted with 2.5 µM of C11-BODIPY staining solution in PBS and then harvested by means of a 0.05% trypsin solution. Subsequently, the supernate was taken out of the fresh medium and immediately assessed using a flow cytometer.

### 3.3. Lactate Dehydrogenase (LDH) and CCK-8 Determination

The determination of LDH and CCK-8 was conducted using the method used in [8]. The method utilized a 96-well plate to seed H9c2 cells at 1 × 10^4^ cells/well. These H9c2 cells were subsequently classified into the above mentioned four groups and were then treated by the method. However, the incubation period was 11 h for the LDH determination. Then, the LDH release reagent was added with the maximum enzyme activity to each group for 1 h. Subsequently, the plate was centrifuged for 5 min at 400× *g*. A volume of 120 µL was removed from each well and added to another blank 96-well plate, respectively. The LDH detection solution (60 µL) was added into each well before incubating the plate for an additional 30 min. The A_490nm_ was determined by a Bio-Kinetics reader (PE-1420; Bio-Kinetics Corporation, Sioux Center, IA, USA). For the CCK-8 determination, the cells incubated for 11 h were treated with 90 µL of RASMX-90011 and 10 µL of CCK-8 solution for 2 h.

### 3.4. Characterization of Mitochondrial ROS in Ferroptotic H9c2 Cells

H9c2 cells were seeded at 1 × 10^5^ cells/well into 12-well plates and then cultured for 24 h. The cultured H9c2 cells were classified into four groups, i.e., control, model, positive control, and sample. The H9c2 cells in the control group were incubated for 12 h in the basal medium. Those in the model group, positive control, and sample groups, however, were incubated with erastin (10 µM) to create ferroptotic damage. Then, the mixture of erastin and medium was removed. The model group, however, was further incubated for 12 h in the above basal medium without the sample solution, while the sample group was incubated for 12 h in the above basal medium with the sample solution. The sample solutions, however, were at different concentrations. Lastly, the positive control was incubated for 12 h in the same medium with 1.0 µM of ferrostatin-1 (Fer-1). To characterize the mitochondrial ROS concentration, the H9c2 cells in the above four groups were incubated with 4′,6-diamidino-2-phenylindole (DAPI) staining. Following a 30 min incubation period, mitochondrial ROS were assayed using the H_2_DCFDA probe (5 µM) and a mitochondrial fluorescent probe [53].

### 3.5. Flow Cytometry for Determination of the Total Intracellular ROS Using a H2DCFDA Probe

The H9c2 cells in the above four groups were used to determine the total intracellular ROS concentration using flow cytometry and an H2DCFDA probe, according to the previous method [54]. Briefly, H9c2 cells were rinsed using PBS two times, and subsequently interacted with 5 µM of H2DCFDA staining solution in PBS at 37 °C for 30 min. The stained H9c2 cells were collected with a 0.05% trypsin solution and then suspended in fresh medium. The supernate was instantly examined by means of a flow cytometer.

### 3.6. PTIO^•^-Scavenging Assay

The free radical scavenging ability of MOA in an aqueous medium was assessed by a PTIO^•^-scavenging assay [55]. Briefly, MOA was dissolved in methanol to prepare the test sample solution (5 mg/mL). The test sample solution was then mixed with a PTIO^•^ aqueous solution (7.4 mmol/L) in different volumes. Thereafter, the sample solutions with different volumes were added to the PTIO^•^ solutions and incubated for 2 h in a phosphate buffer (pH 7.4, 50 mM) and were then measured at a 557 nm wavelength. The PTIO^•^-scavenging percentage was therefore calculated as Equation (1):

(1) $$\mathrm{PTIO}•\;\mathrm{scavenging}\;\%=\frac{A_{0}-A}{A_{0}}\times 100\%$$

where *A*_0_ is the absorbance of the control (reaction system without sample) at 557 nm; and *A* is the absorbance of the reaction mixture with the sample at 557 nm. Methanol was used as the blank group. The above experimental protocols were repeated using Trolox and Fer-1 to replace MOA.

### 3.7. ABTS^•+^-Scavenging Assay

The free radical scavenging ability of MOA in a lipid medium was assessed by an ABTS^+•^-scavenging assay [56], with some modifications. In brief, MOA was dissolved in methanol to prepare the test sample solution (5 mg/mL). The test sample solution was then mixed with different volumes of an ABTS^+•^ solution to establish the dose–response curves. The ABTS^+•^ solution, however, was produced by mixing 700 μL of an (NH_4_)_2_ABTS aqueous solution (7.4 mmol/L) with 700 μL of a K_2_S_2_O_8_ aqueous solution (2.6 mmol/L). The mixture was then incubated in a dark room for 12 h. Thereafter, the incubated mixture was diluted with methanol (for a ratio of approximately 1:15) so that its absorbance at 734 nm was measured to be 0.65 ± 0.01 using a microplate reader (Multiskan FC, Thermo Scientific, Shanghai, China). Then the sample solutions with different volumes were added into the ABTS^+•^ solutions and incubated for 6 min. Thereafter, these mixed solutions were measured at a 734 nm wavelength to acquire the absorbance value using the above microplate reader. The ABTS^+•^-scavenging percentage was therefore calculated as Equation (2):

(2) $$\mathrm{Scavenging}\;\%=\frac{A_{0}-A}{A_{0}}\times 100\%$$

where *A*_0_ is the absorbance of the control (reaction system without sample) at 734 nm; and *A* is the absorbance of the reaction mixture with the sample at 734 nm. Methanol was used as the blank group. The above experimental protocols were repeated using Trolox and Fer-1 to replace MOA.

### 3.8. Molecular Docking Simulation, Molecular Dynamics Simulation, and Molecular Mechanics with Generalized Born and Surface Area Solvation (MM/PBSA) Calculations

The chemical structures of MOA and Fer-1 were obtained from the PubChem database (https://pubchem.ncbi.nlm.nih.gov/, accessed on 14 August 2024) and optimized using the Gaussian 16 C.01 program under the level of B3LYP-D3BJ/6-31G(d,p) [57,58,59,60,61,62]. The absence of an imaginary frequency ensured that the optimized structures were at the local minimum. The crystal structure of each target was obtained from the RCSB Protein Data Bank (https://rcsb.org/, accessed on 29 August 2024) and subjected to preparation processes (i.e., protein extraction, dehydration, hydrogenation, and charge calculation) using AutoDockTools 1.5.7 [63]. The coordinates of the original ligand were taken as a grid center. For targets without original ligands, activity pockets were predicted using PocketMiner (https://pocketminer.azurewebsites.net/, accessed on 29 August 2024) [64], and for targets without experimental structures, structures were predicted using Alphafold (https://alphafoldserver.com/, accessed on 29 October 2024) [65]. The AutoDock Vina 1.2.0 program was employed to perform molecular docking, with the specific parameters shown in Table 3 [66].

Based on the docked structures of the target–ligand complexes, the molecular dynamics simulation was performed using the Gromacs 2020.6 package [67]. The topology parameter of the enzymes was created based on the Amber99SB-ILDN force field, and the topology parameters of the ligands were obtained from the Sobtop 1.0 program (http://sobereva.com/soft/Sobtop/, accessed on 7 November 2024). The Multiwfn 3.8 program was used to derive restrained electrostatic potential (RESP) atomic charges for the ligands based on high-quality electronic wave functions produced by quantum chemical calculations [68]. Then, the topology file of the protein–ligand complex was generated. The complex system was immersed in a closed water box and neutralized through the addition of Na^+^ or Cl^−^ counter-ions. Energy minimization was conducted to remove bad contacts and steric clashes. Subsequently, NVT and NPT equilibrations were performed at a constant temperature of 300 K. Finally, a 20 ns production molecular dynamics run was executed with a target temperature of 300 K and a pressure of 1 atm, and the coordinates were recorded at every 1 ps time step for trajectory analysis. The assistant analysis of trajectories was performed using the Xmgrace and Origin 2021 programs.

The binding free energy ($\Delta G_{\mathrm{bind}}$) of the protein–ligand system was calculated based on an MM/GBSA assay, which was performed using the gmx_MMPBSA program [69]. As for each system, 50 snapshots were extracted, on average, from the 19~20 ns molecular dynamics trajectory for calculation. The energy contributions are further broken into $\Delta G_{\mathrm{gas}}$ and $\Delta G_{\mathrm{solv}}$.

$$\Delta G_{\mathrm{bind}}=\Delta G_{\mathrm{complex}}-\left(\Delta G_{\mathrm{receptor}}+\Delta G_{\mathrm{ligand}}\right)=\Delta H-T\Delta S\approx \Delta E_{\mathrm{MM}}+\Delta G_{\mathrm{solv}}\;\Delta E_{\mathrm{MM}}=\Delta E_{bonded}+\Delta E_{\mathrm{nobonded}}=\left(\Delta E_{bond}+\Delta E_{angle}+\Delta E_{dihedral}\right)+\left(\Delta E_{ele}+\Delta E_{vdW}\right)\;\Delta G_{\mathrm{solv}}=\Delta G_{polor}+\Delta G_{\mathrm{no}\text{-}\mathrm{polor}}\approx \Delta E_{bond}+\Delta E_{surf}$$

Here, $\Delta G_{\mathrm{gas}}$ is the interaction energy and is obtained after summing the internal (bonded) components ($\Delta E_{bond}+\Delta E_{angle}+\Delta E_{dihedral}$) and the non-bonded components ($\Delta E_{ele}+\Delta E_{vdW}$). For $\Delta G_{\mathrm{solv}}$, the polar and non-polar contributions are $\Delta E_{GB}$ (or $\Delta E_{PB}$) and $\Delta E_{surf}$, respectively.

### 3.9. Statistical Analysis

Each antioxidant evaluation experiment was performed in triplicate, and each experiment of quantitative assessment was performed in duplicate. Data are shown as the means ± standard deviations (SDs) of the three independent measurements.

## 4. Conclusions

Methylophiopogonanone A can inhibit ferroptosis in H9c2 cells. This inhibitory effect is mainly attributed to its interaction with enzymes rather than its antioxidant ability (i.e., directional free radical scavenging). The *meta*-di-OHs may be responsible for this weak antioxidant ability. During interactions with enzymes, H-bonding plays a minor role, while hydrophobic interactions play a major role. The hydrophobic interactions mainly depend on non-polar substituent groups, including –CH_3_ *groups*, 3′,4′-*methylenedioxyl* groups, and 9–CH_2_– groups. The latter, however, also serves as a locator to facilitate MOA’s access to enzymes. All these factors contribute to the potential development of MOA and its derivatives as new drugs for the treatment of cardiovascular diseases.

## Figures and Tables

**Table 1:** The binding energy (kcal/mol) of MOA and Fer-1 to nine enzymes using molecular docking simulation.

Enzyme	Binding Energy of MOA	Binding Energy of Fer-1
SOD	−6.5	−5.4
GPX4	−4.0	−4.7
DHODH	−10.1	−8.1
FPN	−8.2	−6.3
FSP1	−8.0	−7.8
NADPH	−9.4	−8.0
SLC7A11	−7.1	−5.7
NRF2	Unavailable	Unavailable
FTH1	Unavailable	Unavailable

**Table 2:** The $\Delta G_{\mathrm{bind}}$ values (kcal/mol) of the MM/PBSA calculation of MOA and Fer-1 to seven enzymes in a molecular dynamics simulation.

System Name	$\Delta E_{bond}$	$\Delta E_{angle}$	$\Delta E_{dihedral}$	$\Delta E_{ele}$	$\Delta E_{vdW}$	$\Delta E_{GB}$	$\Delta E_{surf}$	Total
SOD and Fer_1	0.00 ± 1.14	0.00 ± 2.11	0.00 ± 1.53	−0.96 ± 2.73	−0.18 ± 1.11	2.48 ± 2.55	0.00 ± 0.01	1.34 ± 3.90
SOD and MOA	0.00 ± 1.60	0.00 ± 0.68	0.00 ± 1.13	−54.92 ± 0.55	−17.37 ± 0.22	70.95 ± 1.08	−2.48 ± 0.02	−3.82 ± 1.23
GPX4 and Fer_1	0.00 ± 2.24	0.00 ± 1.75	0.00 ± 1.00	−25.29 ± 0.54	−15.88 ± 0.47	42.95 ± 2.17	−1.91 ± 0.04	−0.12 ± 2.28
GPX4 and MOA	0.00 ± 1.30	0.00 ± 1.81	0.00 ± 2.37	−49.11 ± 0.07	−13.22 ± 0.77	46.57 ± 2.39	−2.14 ± 0.02	−17.90 ± 2.52
DHODH and Fer_1	0.00 ± 1.50	0.00 ± 0.77	0.00 ± 1.29	−62.67 ± 2.17	−30.30 ± 0.24	59.71 ± 2.85	−4.99 ± 0.00	−38.25 ± 3.59
DHODH and MOA	0.00 ± 1.77	0.00 ± 1.91	0.00 ± 0.76	−41.08 ± 1.62	−47.53 ± 0.10	63.67 ± 1.22	−5.68 ± 0.06	−30.63 ± 2.03
FPN and Fer_1	0.00 ± 1.17	0.00 ± 0.71	0.00 ± 1.12	−153.82 ± 1.93	−2.19 ± 0.58	65.47 ± 1.66	−4.26 ± 0.01	−94.80 ± 2.61
FPN and MOA	0.00 ± 2.25	0.00 ± 2.27	0.00 ± 1.85	−17.36 ± 1.36	−38.60 ± 0.80	48.63 ± 1.26	−4.81 ± 0.01	−12.14 ± 2.02
FSP1 and Fer_1	0.00 ± 1.80	0.00 ± 1.56	0.00 ± 1.13	−119.96 ± 2.67	−18.05 ± 0.65	74.53 ± 2.47	−4.84 ± 0.02	−68.32 ± 3.70
FSP1 and MOA	0.00 ± 2.25	0.00 ± 2.27	0.00 ± 1.85	−17.36 ± 1.36	−38.60 ± 0.80	48.63 ± 1.26	−4.81 ± 0.01	−12.14 ± 2.02
NADPH and Fer_1	0.00 ± 2.27	0.00 ± 0.82	0.00 ± 1.11	−136.37 ± 0.20	−24.91 ± 0.58	97.85 ± 1.66	−4.99 ± 0.01	−68.43 ± 1.77
NADPH and MOA	0.00 ± 2.10	0.00 ± 1.48	0.00 ± 1.33	−57.87 ± 2.42	−42.39 ± 0.53	86.46 ± 2.05	−4.98 ± 0.01	−18.78 ± 3.22
SLC7A11 and Fer_1	0.00 ± 1.42	0.00 ± 4.45	0.00 ± 1.55	−89.79 ± 2.68	−28.67 ± 0.41	61.66 ± 3.63	−5.22 ± 0.01	−62.02 ± 4.52
SLC7A11 and MOA	0.00 ± 2.50	0.00 ± 3.07	0.00 ± 1.96	−36.54 ± 0.79	−30.31 ± 0.41	58.20 ± 0.26	−4.51 ± 0.00	−13.16 ± 0.93

Note: MM/PBSA, molecular mechanics with Poisson–Boltzmann and surface area solvation.

**Table 3:** The main parameters for molecular docking and the molecular dynamics simulation.

Target Names	PDB Codes	Pocket Coordinates	Pocket Points	Spacing	Docking Runs
SOD	1PU0	67.79, 49.86, 12.09	40 × 40 × 40	0.5	100
GPX4	5H5Q	3.28, 24.73, −18.10			
NADPH	2CDU	2.00, −1.25, −0.30			
FSP1	8WIK	15.62, −4.96, −21.41			
DHODH	6LP7	−33.29, −11.83, 1.31			
DMT1	4YJ0	−29.95, −17.72, −26.31			
FTH1	4OYN	37.10, 19.79, 29.48			
FPN	8C03	117.51, 120.35, 121.50			
SLC7A11	7EPZ	148.71, 147.14, 119.97			

## Data Availability

All the data used to support the findings of this study are available from the corresponding author upon reasonable request.
